# Patient Satisfaction With Quality of Care at the Kingdom of Saudi Arabia

**DOI:** 10.7759/cureus.32102

**Published:** 2022-12-01

**Authors:** Afnan Almass, Hanan M Aljohani, Rayyan M Alhaqbani, Aroob M Alromih, Shahd Hadal, Hesham S Abozaid

**Affiliations:** 1 Emergency Medicine, Ministry of Health, Riyadh, SAU; 2 Medicine, Imam Mohammad Ibn Saud Islamic University, Riyadh, SAU

**Keywords:** saudi arabia, quality of care, emergency medicine, satisfaction, patient

## Abstract

Background

The emergency department (ED) is the first contact of many individuals who require acute health services. This study was the first to be conducted among patients in different regions of Saudi Arabia to determine patient satisfaction with emergency healthcare services using the Arabic version of the Echelle de Qualité des Soins en Hospitalisation (EQS-H).

Methodology

This cross-sectional study was conducted among 2,997 patients admitted to the ED in different hospitals in different regions of Saudi Arabia. The study was based on a self-reported questionnaire validated to assess the satisfaction of patients with ED healthcare services called EQS-H. In this study, we used an Arabic version of the questionnaire. Statistical analyses were performed using R version 3.6.3.

Results

The study was conducted among 2,997 patients (36.7% males and 63.3% females). Regarding region, respondents from the central region represented one-third of the sample (31.7%), followed by respondents from the western, eastern, and southern regions (24.1%, 16.9%, and 14.6%, respectively). Statistical analysis showed that the average percentage score for the clarity of information was significantly higher in the central region than in other regions and was lowest in the eastern region. Individuals aged 26-35 years (B = -2.54 and P < 0.05), male sex (B = -1.63 and P < 0.05), Saudis (B = -3.81 and P < 0.05), longer ED length of stay (LOS) (B = -2.19 and P < 0.001), worse perceived health state, and lower life satisfaction scale scores were significantly associated with lower levels of satisfaction with ED services. Perceived improvement is the strongest predictor of satisfaction.

Conclusion

Moderate satisfaction levels were reported in both the clarity of information domain and relationship with staff domain among patients admitted to EDs in different regions of Saudi Arabia, with better results in the central region.

## Introduction

There are a growing number of efforts to compare the service quality of healthcare organizations based on patient satisfaction data. Such efforts inevitably raise questions regarding the fairness of the comparisons. Fair comparisons presumably should not penalize (or reward) healthcare organizations for factors that influence satisfaction scores but are not under the control of managers or clinicians. Based on previous research, these factors might include the demographic characteristics of patients (e.g., age) and the institutional characteristics (e.g., size) of the healthcare organizations where care was received [1].

Patient satisfaction is a measure of the extent to which patients are content with the healthcare they receive from their healthcare provider. Patient satisfaction is an important factor in determining the success of a healthcare facility [2]. It is an individual’s cognitive evaluation of and emotional reaction to their healthcare experience. Modifiable factors that contribute to satisfaction include physician-patient communication, setting appropriate expectations, minimizing waiting times, and providing continuity of care. Since emergency care is often the first port of call for people in need of acute care, the level of satisfaction with emergency care can serve as a measure of its quality [3]. This study aimed to assess the level of patient satisfaction in the emergency department (ED) and determine the factors that affect patient satisfaction in the emergency department.

## Materials and methods

We conducted a cross-sectional study to assess the level of patient satisfaction in the emergency department and to determine the factors that lead to patient satisfaction in the emergency department in Saudi Arabia. The study design was approved by the Institutional Review Board of Imam Mohammad Ibn Saud Islamic University. The study population consisted of the Saudi Arabian population aged 18 years and older. Data were collected through an online questionnaire using social media and administered to the population of the Kingdom of Saudi Arabia regarding the factors that affect their satisfaction in the emergency department. Participants less than 18 years old were excluded. Informed consent was obtained from all participants. The required sample size was calculated using the Epidemiological Information Package (EPI INFO) (Centers for Disease Control and Prevention, Atlanta, GA) version 7.2. According to the software, Saudi Arabia’s population who are 18 years and older is 26,456,921, and the sample size needed is 385 participants, at least for each region of Saudi Arabia, using a margin of error of ±5%, a confidence level of 95%, and a 50% expected frequency.

Questionnaire

Data contained multi-item questionnaires adopted from a previously published study conducted in King Abdulaziz Medical City, and approval to use the questionnaires was obtained [4]. The Arabic version of the Echelle de Qualité des Soins en Hospitalisation (EQS-H) was used to assess patients’ satisfaction with the quality of medical and nursing care, which is a self-reported questionnaire that includes 15 items related to two domains of patient satisfaction. Five-point Likert scale items were used to assess patients’ satisfaction, with higher scores indicating higher satisfaction: 1 = poor, 2 = moderate, 3 = good, 4 = very good, and 5 = excellent. The overall satisfaction score is the sum of the scores for each item. The domain scores for the clarity of information (five items) and relationships with the emergency care center (ECC) staff (nine items) were also calculated. Six items were related to the quality of the medical information domain, and 10 were related to the relationship with staff and the daily routine domain. The quality of the medical information domain included the following six items: the clarity of information about the symptoms, reasons for the investigations, results of the investigation, reasons for the given medications, side effects of those medications, and safety procedures provided at discharge. The domain scores range from six to 30 points. The relationship with the ECC staff domain (nine questions) included the following ten questions: knowing the treating physician, providing privacy, department services (food, dressing, and cleanliness), analgesia, the response of the nursing staff, organization in the section, the level of understanding within the department staff, time given by the nursing staff, medical decision sharing, care, and treatment in general. However, some modifications were made in the current study. The knowledge of the treating physician was included as a yes/no question and not in the scoring. The extent of participation in medical decision-making was included as a two-part question. First, the respondents were asked whether they had participated in the study. Only those who responded with yes were asked about the extent of their participation. Thus, those who did not participate were awarded one point for the second question. The total score for this domain ranged from nine to 45 points.

Statistical analysis

Data are expressed as the mean percentage score for both domains and the overall score. Statistical analyses were performed using R version 3.6.3. Counts and percentages were used to summarize the distribution of categorical variables, and mean ± SD was used to summarize continuous variables. Pearson’s chi-square test and chi-square test for linear trends were used to assess the association between categorical variables. Student’s t-test and analysis of variance (ANOVA) were used to assess the association between the sociodemographic characteristics of the patients and continuous normal outcomes. The Mann-Whitney and Kruskal-Wallis tests were used for non-normal variables. Pearson’s correlation was used to assess the associations between continuous variables. Multiple linear regression analysis was used to assess the predictors of patient satisfaction scores. Independent variables included sex, age, region, nationality, and waiting time in the emergency department (ED). Other predictors included the perception of health status compared to people of the same age, life satisfaction (score of 1-10), and perceived improvement compared to admission. Statistical significance was set at P < 0.05.

## Results

The sample included responses from 2,997 patients (36.7% males and 63.3% females). The respondents aged 18-25 years represented approximately half of the sample, while respondents aged 26-35 years represented one-quarter. Respondents older than 55 years represented only 5%. More than half of the patients (54.4%) were single, and 40.3% were married.

Regarding region, respondents from the central region represented one-third of the sample (31.7%), followed by respondents from the western, eastern, and southern regions (24.1%, 16.9%, and 14.6%, respectively). The length of stay (LOS) in the ED ranged from <30 minutes (16.3%) to 6-9 hours (5.34%), with less than one-half of the patients spending 30-120 minutes (41.3%). Hospital length of stay (LOS) ranged from <1 day (45%) to >2 days (35.2%). One-quarter of the respondents required admission (24%). One-half of the respondents thought their health state was similar to those in the same age group (51.9%), and more than one-third perceived it as better (38.5%). Only 9.64% perceived their health state as worse. The average overall life satisfaction (OLS) was 8.04 ± 2.18. Most respondents reported improved health compared with admission (~85%), and only 13.2% reported no improvement (Table 1).

**Table 1:** Descriptive statistics for the study sample ED: emergency department

	All	N
	N=2,997	
Gender		2,997
Female	1,898 (63.3%)	
Male	1,099 (36.7%)	
Age		2,997
18-25	1,384 (46.2%)	
26-35	623 (20.8%)	
36-45	456 (15.2%)	
46-55	387 (12.9%)	
56-65	121 (4.04%)	
>65	26 (0.87%)	
Nationality		2,997
Non-Saudi	142 (4.74%)	
Saudi	2,855 (95.3%)	
Region		2,997
Central region	951 (31.7%)	
Eastern region	505 (16.9%)	
Northern region	382 (12.7%)	
Southern region	438 (14.6%)	
Western region	721 (24.1%)	
Length of stay (LOS) in the ED		2,997
<30 minutes	490 (16.3%)	
>9 hours	189 (6.31%)	
2-4 hours	640 (21.4%)	
30-120 minutes	1,238 (41.3%)	
4-6 hours	280 (9.34%)	
6-9 hours	160 (5.34%)	
Hospital LOS		1,134
<1 day	510 (45.0%)	
1-2 days	225 (19.8%)	
>2 days	399 (35.2%)	
Did you require admission?		2,997
No	2,278 (76.0%)	
Yes	719 (24.0%)	
Health state compared with others in the same age group		2,997
1: Worse	289 (9.64%)	
2: No difference	1,554 (51.9%)	
3: Better	1,154 (38.5%)	
Improvement compared with admission		2,997
1: No improvement	395 (13.2%)	
2: Little improvement	1,392 (46.4%)	
3: Improved a lot	1,210 (40.4%)	
Life satisfaction (out of 10)	8.04±2.18	2,997

The analysis showed that age, sex, and nationality varied significantly between the regions. The proportion of males attending the ED was lowest in the southern region and highest in the northern region. This percentage was not significantly different among the remaining three regions. Respondents from the eastern region were somewhat older than those from the remaining regions, while respondents from the southern region were the youngest. Non-Saudi respondents were more prevalent in the western region (9.57%) than in other regions. There was a trend toward increasing LOS in the ED in the central region. Hospital LOS was lowest in the southern region. The requirements for admission, perceived health status and life satisfaction, were not significantly different between the regions. Similarly, the perceived improvement in health compared to admission was not significantly different between the states (P = 0.161) (Table 2).

**Table 2:** Comparison of sociodemographic characteristics between regions Statistical analysis was performed using the chi-square test of independence *Significant at P < 0.05 ED: emergency department

	Central region	Eastern region	Northern region	Southern region	Western region	P-value
	N=951	N=505	N=382	N=438	N=721	
Gender						<0.001*
Female	603 (63.4%)	306 (60.6%)	189 (49.5%)	355 (81.1%)	445 (61.7%)	
Male	348 (36.6%)	199 (39.4%)	193 (50.5%)	83 (18.9%)	276 (38.3%)	
Age						<0.001*
18-25	466 (49.0%)	123 (24.4%)	201 (52.6%)	271 (61.9%)	323 (44.8%)	
26-35	173 (18.2%)	113 (22.4%)	64 (16.8%)	70 (16.0%)	203 (28.2%)	
36-45	122 (12.8%)	138 (27.3%)	60 (15.7%)	41 (9.36%)	95 (13.2%)	
46-55	139 (14.6%)	97 (19.2%)	50 (13.1%)	39 (8.90%)	62 (8.60%)	
56-65	40 (4.21%)	33 (6.53%)	6 (1.57%)	14 (3.20%)	28 (3.88%)	
>65	11 (1.16%)	1 (0.20%)	1 (0.26%)	3 (0.68%)	10 (1.39%)	
Nationality						<0.001*
Non-Saudi	38 (4.00%)	13 (2.57%)	10 (2.62%)	12 (2.74%)	69 (9.57%)	
Saudi	913 (96.0%)	492 (97.4%)	372 (97.4%)	426 (97.3%)	652 (90.4%)	
Length of stay (LOS) in the ED						<0.001*
<30 minutes	127 (13.4%)	83 (16.4%)	88 (23.0%)	104 (23.7%)	88 (12.2%)	
>9 hours	76 (7.99%)	25 (4.95%)	19 (4.97%)	25 (5.71%)	44 (6.10%)	
2-4 hours	212 (22.3%)	107 (21.2%)	74 (19.4%)	84 (19.2%)	163 (22.6%)	
30-120 minutes	383 (40.3%)	210 (41.6%)	152 (39.8%)	181 (41.3%)	312 (43.3%)	
4-6 hours	98 (10.3%)	50 (9.90%)	32 (8.38%)	28 (6.39%)	72 (9.99%)	
6-9 hours	55 (5.78%)	30 (5.94%)	17 (4.45%)	16 (3.65%)	42 (5.83%)	
Hospital LOS						0.020*
<1 day	155 (43.1%)	71 (39.9%)	60 (42.9%)	95 (55.2%)	129 (45.4%)	
1-2 days	71 (19.7%)	35 (19.7%)	40 (28.6%)	26 (15.1%)	53 (18.7%)	
>2 days	134 (37.2%)	72 (40.4%)	40 (28.6%)	51 (29.7%)	102 (35.9%)	
Did you require admission?						0.748
No	715 (75.2%)	383 (75.8%)	290 (75.9%)	344 (78.5%)	546 (75.7%)	
Yes	236 (24.8%)	122 (24.2%)	92 (24.1%)	94 (21.5%)	175 (24.3%)	
Health state compared with the same age group						0.304
1: Worse	86 (9.04%)	46 (9.11%)	36 (9.42%)	49 (11.2%)	72 (9.99%)	
2: No difference	504 (53.0%)	266 (52.7%)	190 (49.7%)	203 (46.3%)	391 (54.2%)	
3: Better	361 (38.0%)	193 (38.2%)	156 (40.8%)	186 (42.5%)	258 (35.8%)	
Improvement compared with admission						0.161
1: No improvement	122 (12.8%)	72 (14.3%)	49 (12.8%)	69 (15.8%)	83 (11.5%)	
2: Little improvement	416 (43.7%)	231 (45.7%)	192 (50.3%)	204 (46.6%)	349 (48.4%)	
3: Improved a lot	413 (43.4%)	202 (40.0%)	141 (36.9%)	165 (37.7%)	289 (40.1%)	
Life satisfaction	8.16 (2.14)	7.95 (2.00)	7.98 (2.52)	7.97 (2.26)	8.02 (2.10)	0.318

**Figure 1:**
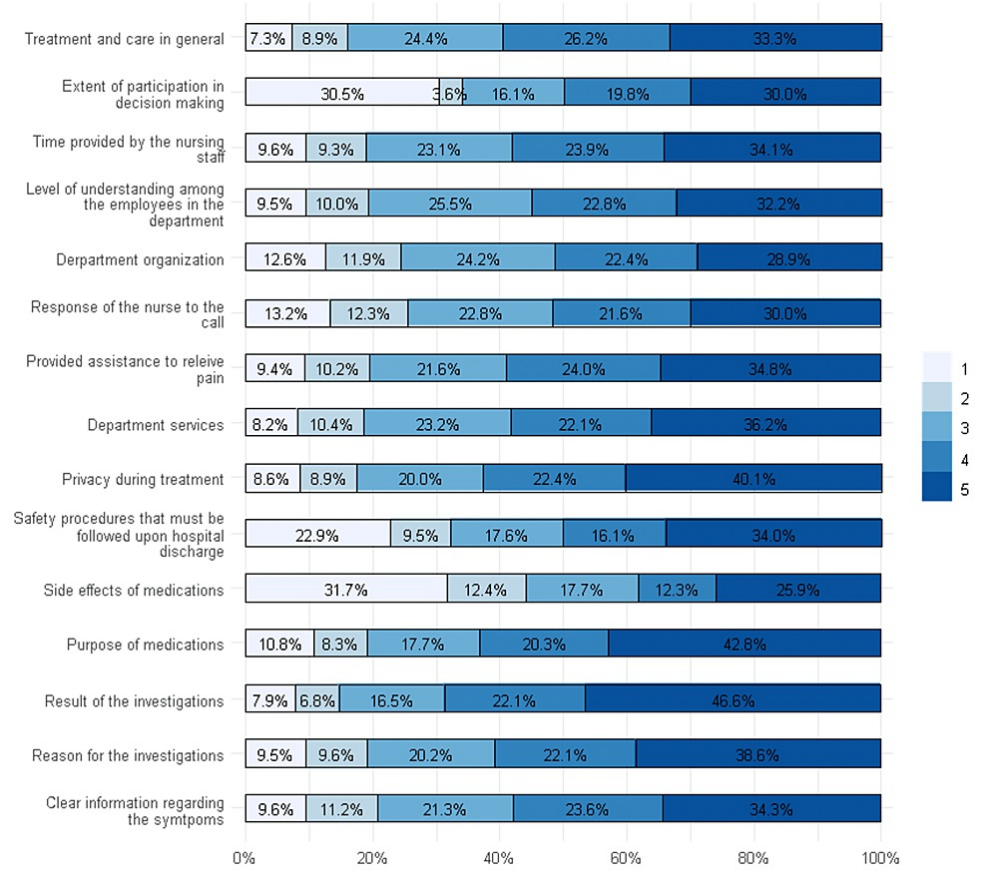
Responses to Echelle de Qualité des Soins en Hospitalisation (EQS-H) domains of patient satisfaction to emergency care

Results showed that 31.7% of the respondents were poorly satisfied with the clarity of the side effects of medications, and 30.5% were poorly satisfied with the extent of participation in decision-making. The highest satisfaction was observed in the results of the investigations and the purpose of the medications. One-quarter of the respondents were poorly satisfied with the clarity of the safety procedures that must be followed upon discharge (Figure 1).

A statistically significant difference was observed between regions across all items of satisfaction. Statistical analysis showed that the average percentage score for the clarity of information was significantly higher in the central region than in other regions and was lowest in the eastern region. The same pattern was observed for the relationship between the staff and overall scores (Table 3).

**Table 3:** Association between the region and EQS-H score Analysis was performed using the Kruskal-Wallis test. Data are presented as mean percentage score *Significant at P < 0.05 EQS-H: Echelle de Qualité des Soins en Hospitalisation

	Central region	Eastern region	Northern region	Southern region	Western region	P-value
	N=951	N=505	N=382	N=438	N=721	
Clarity of information	77.0 (57.0; 97.0)	67.0 (50.0; 87.0)	73.0 (53.0; 93.0)	73.0 (57.0; 90.0)	73.0 (53.0; 90.0)	<0.001*
Relationship with staff	78.0 (58.0; 96.0)	69.0 (56.0; 87.0)	73.0 (56.0; 91.0)	71.0 (53.8; 87.0)	71.0 (56.0; 89.0)	<0.001*
Overall score	77.0 (59.0; 93.0)	68.0 (55.0; 85.0)	72.0 (56.0; 89.0)	72.0 (55.2; 87.0)	72.0 (55.0; 88.0)	<0.001*

Respondents aged 26-35 years showed lower satisfaction with the clarity of information (B = -3.62 and P < 0.05), relationship with staff (B = -1.84 and P < 0.1), and overall satisfaction (B = -2.54 and P < 0.05). Males had significantly lower satisfaction with the clarity of information than females (B = -1.63 and P < 0.05). Saudis showed lower satisfaction with the clarity of information (B = -3.07 and P = 0.05), relationship with staff (B = -4.35 and P < 0.05), and overall satisfaction (B = -3.81 and P < 0.05). Satisfaction was significantly higher in the central region than in the other regions (P < 0.05). A longer ED LOS was associated with lower satisfaction with the clarity of information (B = -1.88 and P < 0.001), relationship with staff (B = -2.4 and P < 0.001), and overall satisfaction (B = -2.19 and P < 0.001). A better perceived health state was associated with higher satisfaction with the clarity of information (B = 4.46 and P = 0.001), relationship with staff (B = 4.36 and P = 0.001), and overall satisfaction (B = 4.39 and P < 0.001). A higher score on the life satisfaction scale was associated with higher satisfaction in the two domains of the questionnaire and overall satisfaction score. Perceived improvement is the strongest predictor of satisfaction. Respondents who reported little improvement reported a higher satisfaction score of 16 points than patients who did not report any improvement. Patients who improved reported a satisfaction score that was higher by ~30 points compared to patients who did not report any improvement (Table 4).

**Table 4:** Factors associated with overall satisfaction, satisfaction with the clarity of information, and satisfaction with the relationship with staff Length of stay in the emergency department (ED) was included as a continuous variable to assess the association between the increase in this variable and satisfaction *Significant at P < 0.05 CI: confidence interval; LOS: length of stay

	Clarity of information			Relationship with staff			Overall		
Predictors	Estimates	CI	P-value	Estimates	CI	P-value	Estimates	CI	P-value
Age									
18-25	Reference			Reference			Reference		
26-35	-3.62	-5.92 to -1.33	0.002	-1.84	-3.96-0.28	0.090	-2.54	-4.59 to -0.50	0.015*
36-45	-1.80	-4.78-1.18	0.237	-0.43	-3.19-2.32	0.759	-0.99	-3.65-1.66	0.464
46-55	-2.29	-5.53-0.96	0.167	-0.83	-3.83-2.17	0.589	-1.39	-4.28-1.50	0.345
56-65	-2.74	-7.30-1.83	0.240	-0.92	-5.15-3.30	0.669	-1.68	-5.75-2.38	0.417
>65	-2.95	-11.12-5.22	0.479	0.09	-7.47-7.64	0.982	-1.15	-8.43-6.13	0.757
Gender: male versus female	-1.63	-3.18 to -0.07	0.041	-0.86	-2.30-0.59	0.245	-1.16	-2.55-0.23	0.102
Region									
Central region									
Eastern region	-4.59	-6.77 to -2.42	<0.001	-4.34	-6.36 to -2.33	<0.001	-4.44	-6.38 to -2.50	<0.001*
Northern region	-2.79	-5.13 to -0.45	0.020	-3.00	-5.17 to -0.83	0.007	-2.90	-4.99 to -0.81	0.007*
Southern region	-2.64	-4.89 to -0.39	0.021	-4.85	-6.93 to -2.77	<0.001	-3.96	-5.96 to -1.95	<0.001*
Western region	-3.08	-4.99 to -1.17	0.002	-4.18	-5.95 to -2.41	<0.001	-3.73	-5.44 to -2.03	<0.001*
Hospital LOS	-1.88	-2.42 to -1.34	<0.001	-2.40	-2.89 to -1.90	<0.001	-2.19	-2.67 to -1.71	<0.001*
Perceived health state									
Worse	Reference			Reference			Reference		
No difference	0.13	-2.37-2.63	0.918	1.58	-0.73-3.90	0.179	1.00	-1.23-3.23	0.380
Better	4.46	1.75-7.17	0.001	4.36	1.86-6.87	0.001	4.39	1.98-6.81	<0.001*
Life satisfaction	1.63	1.29-1.97	<0.001	1.71	1.39-2.02	<0.001	1.68	1.38-1.98	<0.001*
Improvement compared with admission									
No improvement	Reference			Reference			Reference		
Little improvement	17.83	15.64-20.03	<0.001	16.22	14.19-18.25	<0.001	16.83	14.88-18.79	<0.001*
Improved a lot	30.87	28.56-33.19	<0.001	31.07	28.93-33.22	<0.001	30.96	28.89-33.03	<0.001*
Observations	2,997			2,997			2,997		
R^2^/R^2^ adjusted	0.318/0.312			0.367/0.361			0.378/0.372		

## Discussion

The ED is the first contact of many individuals who require acute health services. This study was the first to be conducted among patients in different regions of Saudi Arabia to determine patient satisfaction with emergency healthcare services using the Arabic version of the EQS-H. The EQS-H questionnaire was one of the several surveys developed and validated in many countries to measure patients’ satisfaction [5,6]. In this study, the EQS-H questionnaire demonstrated good reliability. Previous studies have evaluated patient satisfaction in Western countries [7,8]. However, little is known about patient satisfaction in Arab countries where sociocultural values are different [9]. In the current study, the mean score of total satisfaction reported among patients from different regions in Saudi Arabia was 72.2, while the clarity of information and relationship with staff mean scores were 72.6 and 72.4, respectively, which correspond to a moderate level of satisfaction. There was a significant difference among regions of Saudi Arabia considering satisfaction levels, where participants in the central region (951) had the highest level of satisfaction, while the eastern region (505) had the lowest level of satisfaction, and the other regions had similar results.

A previous study conducted by Abolfotouh et al. among patients admitted to the emergency department at King Abdulaziz Medical City, Riyadh, Saudi Arabia, showed that the mean overall satisfaction was 70.36 (SD = 17.4), the clarity of information domain was 67.49 (SD = 21.49), and relationship with staff domain was 71.79 (SD = 18.4) [4]. In a recent study conducted by Banjar and Nafisah, the authors reported lower scores for the clarity of information domain and relation with staff domain at 40 and 39.9 [10]. The same study showed a significant difference among different regions, where higher satisfaction was reported in the southern province, followed by the northern, eastern, central, and western provinces [10]. Another study in the western region of Saudi Arabia showed that 48.93% of the patients were highly satisfied with the clarity of information, 33.14% were moderately satisfied, and 17.93% were unsatisfied. Moreover, this study showed that 38.4% were highly satisfied with the relationship with staff and 36.65% were moderately satisfied [11].

Our results showed a significant relationship between perceived improvement in the health status of patients and their satisfaction score, which is in agreement with previous studies [9,12]. Perceived improvement in the health status of patients indicates the relief of suffering from symptoms of medical conditions and should logically be associated with higher satisfaction levels [12]. However, accurate interpretations of comparative satisfaction data require consideration of patients’ profiles of their conditions [13]. In a study conducted in Korea, the authors reported a positive effect of physician empathy on patient satisfaction and compliance, as well as the positive impact of increased compliance on patient health [14]. A previous study showed that patient satisfaction was significantly associated with positive health outcomes of patients and patient-doctor communication [15]. Another study conducted in Italy considered the relationship between disease complications of diabetic patients and patient satisfaction and found that physician empathy was significantly associated with clinical outcomes related to patient satisfaction [16]. The same results were reported in a recent study conducted among 235 patients admitted to the medical ward of an educational tertiary healthcare center in Jeddah, Saudi Arabia, which found that perceived improvement in the health status of the patients was significantly associated with their satisfaction [17]. In this study, the main reasons for visiting the ED were shortness of breath (SOB) (17.8%), followed by sprain/fracture (11.9%), abdominal pain during pregnancy (10.0%), allergy (9.82%), trauma/wound (9.4%), and inflammation (8.65%). In another study, the reasons for visiting the ED were abdominal pain, shortness of breath, vaginal bleeding, and dizziness [4].

Self-perceived health status is not usually considered important in satisfaction studies, particularly when comparing different patient groups [5]. In the current study, perceived health status was significantly associated with satisfaction scores; those who reported having a better health state had a higher satisfaction level than those who reported no difference or worsened health state. A previous study showed that a high level of general satisfaction with life is associated with a positive viewpoint toward satisfaction with care [9]. On the other hand, another study showed that higher levels of satisfaction with life are associated with higher expectations of health services than those with lower levels of satisfaction with life and are associated with a low level of satisfaction with healthcare services [4]. Moreover, the results of the current study showed higher satisfaction scores among female patients than among male participants in both domains, which is consistent with other studies [4,18]. These results may indicate that males have greater expectations than females do. However, other studies have shown that males are more satisfied than females in both domains [5,9,12,19]. Additionally, our results showed that respondents aged 26-35 years showed lower satisfaction with the clarity of information (B = -3.62 and P < 0.05), relationship with staff (B = -1.84 and P < 0.1), and overall satisfaction (B = -2.54 and P < 0.05). Some previous studies showed that age is an essential determinant factor in determining satisfaction levels, where older participants had lower levels of satisfaction [10,11,20-22].

In the current study, we found that poor satisfaction of the participants was related to the clarity of side effects of the medication, the extent of participation in decision-making, and the clarity of safety procedures that must be followed upon discharge, while the highest satisfaction was observed for the results of investigations and the purpose of medications. Similar results were reported in a recent study that showed poor satisfaction was associated with the clarity of possible side effects of the medications and symptoms they should monitor for the future [11]. Moreover, another study conducted by Owaidh et al. showed that 20.1% of the patients were not satisfied with the clarity of possible side effects associated with medications [23]. Other previous studies have shown that the satisfaction of patients with the emergency department is affected by the provision of information, interpersonal relationships between patients and staff, and waiting time [18,24,25]. Furthermore, two previous studies showed poor satisfaction considering the clarity of information, particularly regarding side effects, symptoms, purposes of medication, and reasons for the results of investigations [4,9].

## Conclusions

We found that a moderate satisfaction level was reported in both the clarity of information domain and relationship with staff domain among patients admitted to EDs in different regions of Saudi Arabia, with better results in the central region. We also observed that among the patients, the most common factors associated with their poor satisfaction were the low clarity of side effects of the medication, the extent of participation in decision-making, and the clarity of safety procedures that must be followed upon discharge. This study showed that improving the communication between hospital staff and patients would increase the overall satisfaction of the patients and their outcomes. In order to improve patient satisfaction in the emergency department, more understanding and apperception of these factors are essential. Also, we recommend implanting programs that improve communication skills for healthcare providers. On the other hand, it is important to improve the health education of consumers so that they can have a better understanding of healthcare services. Further studies to examine the physicians’ satisfaction regarding the facilities of hospitals are recommended.
